# A Community Mural Tour: Facilitating Experiential Learning About Social Determinants of Health

**DOI:** 10.5811/westjem.2020.9.48738

**Published:** 2020-11-20

**Authors:** Kamna S. Balhara, Nathan Irvin

**Affiliations:** * Johns Hopkins University School of Medicine, Department of Emergency Medicine, Baltimore, Maryland; † Johns Hopkins Bloomberg School of Public Health, Center for Mental Health and Addiction Policy Research, Baltimore, Maryland

## Abstract

To successfully provide effective patient care within a healthcare system and broader society facing health inequities and social injustice, emergency medicine (EM) residents must demonstrate a nuanced understanding of social determinants of health (SDOH). Classroom or bedside instruction may be insufficient to generate meaningful engagement with patients’ social contexts; experiential collaborative learning with community engagement has been suggested as an ideal modality for education about SDOH. We describe a low-cost, easily replicable activity involving observation and discussion of community murals within built environments. The tour was planned by EM faculty with expertise in graduate medical education, social EM, and the use of art in medical education. Prior to the activity, faculty selected murals situated in a variety of neighborhoods that would spark discussion on SDOH. Over the two-hour tour, residents stopped at city murals on a pre-planned route and engaged in observation and discussion. Faculty facilitators used established arts pedagogy, including visual thinking strategies and the concept of the “third thing,” to facilitate a collaborative exploration of murals, surrounding communities, and larger implications for patients. The activity was successful in providing residents with a nuanced, context-specific approach to SDOH, sparking greater curiosity about the communities they serve, and engaging residents in reflection and conversation about personal preconceptions and how to better engage with surrounding communities. Since murals and street art are present and accessible in many different settings, residency programs could consider implementing a similar activity as part of their didactic curriculum.

## BACKGROUND

For emergency medicine (EM) trainees working within a healthcare system and broader society burdened by health inequity and social injustice, a nuanced understanding of social determinants of health (SDOH) is an essential competency.^1^–^3^ EM trainees are often new to communities in which they work and may have vastly different lived experiences from their patients.^4^ Often caught between disparate realities, EM trainees must excel at anticipating patients’ needs and understanding barriers patients face in order to provide effective and compassionate care, and they need training to do so. Traditional resident education focused largely on classroom- or hospital-based didactics may fail to generate a deeper, context-specific understanding of SDOH. Experiential and collaborative learning, including community engagement, may lead to transformative learning in SDOH.^5^ Expanding beyond hospital walls into communities where patients live may represent an opportunity to better educate residents about SDOH.

We describe an “out-of-the-hospital” approach using city murals to address this gap in education. Art has been widely applied in medical education with impacts on observation skills, critical thinking, and empathy.^6^,^7^ Additionally, it can positively impact preconceptions toward patients and serve as a lens to examine biases.^8^,^9^ Much of art-based education, however, is museum-based, where art is often not representative of the diversity of surrounding communities.^6^, ^10^, ^11^ Conversely, murals are a form of social expression often designed by or in conjunction with community members. Directly embedded into built environments, they evolve as neighborhoods change.^12^ With these elements in mind, as part of a year-long social EM and humanities curriculum, we designed a tour of murals dispersed throughout the city to orient EM trainees to different neighborhoods and their social context.

## OBJECTIVES

Upon tour completion, participants were expected to be able to do the following: 1) demonstrate improved understanding of patients’ built environments and social contexts; 2) engage in reflection and discussion on SDOH impact on patients, with a focus on built environment; and 3) use visual thinking strategies to engage in close looking and perspective-sharing.

## CURRICULAR DESIGN

We planned our tour using an online platform that maps murals in our program’s city. A faculty member with expertise in social EM, a discipline that studies the interaction of social forces and health in EM, chose murals representative of the five categories of SDOH outlined in Healthy People 2020.^13^ Murals were selected both for content and inclusivity of neighborhoods. Background research on works’ artists and relevance to the neighborhood was conducted. We developed a tour route using Google Maps.

The activity was advertised to all residents in our four-year program, although this was a smaller-scale pilot due to space constraints. Residents registered on a first-come, first-serve basis. Three EM faculty with expertise in medical education, art-based education, and social EM jointly facilitated the session for five participants. Observation and discussion of murals was mediated by faculty using visual thinking strategies (VTS) and the “third things” concept. Visual thinking strategies comprise a widely used pedagogical framework that fosters critical thinking, empathy, intellectual curiosity, and openness to the unfamiliar.^14^ In medical education, VTS enhances team cohesion, analytical skills, and communication.^15^

**Figure 1:**
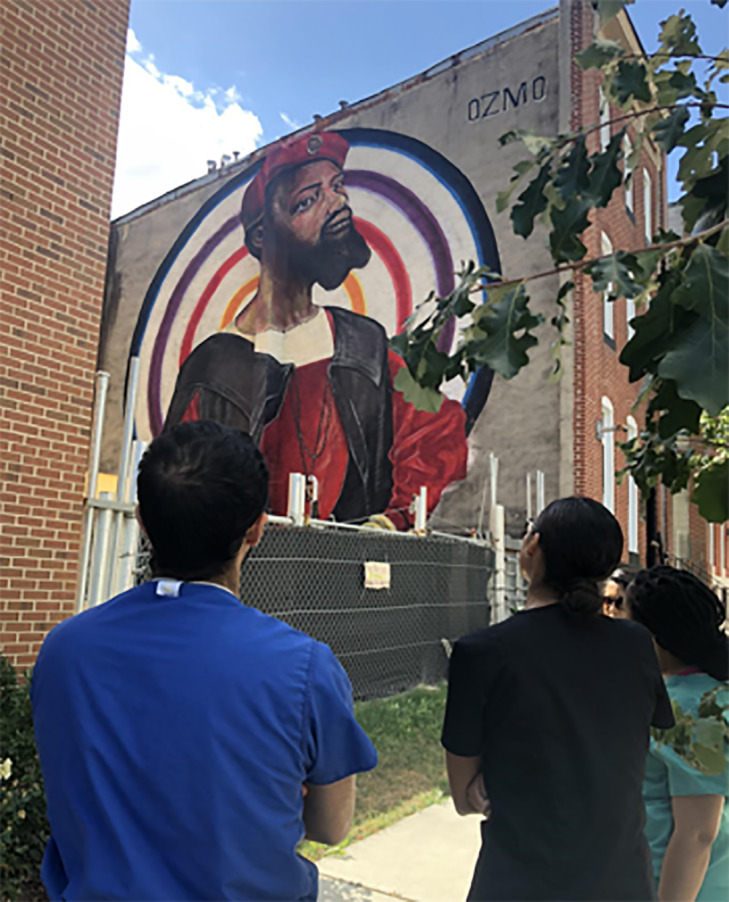
Emergency medicine residents discussing a neighborhood mural.

The tour also framed murals as “third things,” which are reflective triggers or conversational mediators that create safe spaces for perspective-sharing.^16^–^18^ Using these two techniques, learners collaboratively made meaning from what they saw. At each mural, learners disembarked from the van to participate in a facilitated discussion about the work and to reflect on the surrounding built environment (Figure 1). This cycle was repeated over two hours. Further reflection occurred while in transit between sites, and debriefing was conducted as participants were driven back to the hospital.

## IMPACT/EFFECTIVENESS

The tour’s impact was assessed via the richness of discussions during the activity, based upon faculty’s notes on common discussion themes. Participants’ insights were expansive, including observations on the presence of blight and vacancies, varied community assets including religious institutions, and local history as it related to either the community or the murals. Participants exhibited intellectual curiosity about communities visited and a more nuanced understanding of the built environment and role of SDOH in health. As “third things,” murals led participants to share differing perspectives and explore their own biases. Participants concluded by generating their own ideas about meaningful ways they could interact with the community.

Participants completed anonymous, electronic survey-based evaluations and universally rated the experience favorably (4/4 on a Likert scale). Participants’ free-text responses reflected a universal appreciation for the opportunity to both view murals, which they found enlightening, and to experience communities that were new to them. One learner commented they subsequently felt inspired to be more involved in community engagement efforts. Participants reported that the tour generated a desire to learn more about the communities visited. Creating a learning environment where traditional hierarchies were leveled permitted a safe and open discussion among learners from different levels. In response to participants’ feedback, future sessions will include additional neighborhoods and more historical context in partnership with local communities and artists.

Our evaluation was limited by a small number of volunteer participants susceptible to selection bias, and was not designed to capture downstream impacts on knowledge and behavior. However, we believe the tour was an important step toward contextualizing the interplay between social factors and health. Mural tours are a low-cost, replicable approach to experiential and collaborative exposure to SDOH, and represent an opportunity for faculty to expand pedagogic partnerships beyond hospital walls to include local health officials, community members, and artists. Murals can spark context-specific discussion of social issues faced by patients, along with exposure to patient realities that are often invisible in clinical settings. While future studies are needed to evaluate downstream impacts on transfer of residents’ SDOH knowledge to clinical care, this creative approach takes residents outside hospital walls and comfort zones. Such experiences can be invaluable as we move to meet the urgent need to heal some of the deep wounds that exist in our society and institutions.
